# Bacteria may be in the liver, but the jury is still out

**DOI:** 10.1172/JCI158999

**Published:** 2022-04-15

**Authors:** Nichole A. Broderick, Laszlo Nagy

**Affiliations:** 1 Department of Biology, Johns Hopkins University, Krieger School of Arts and Sciences, Baltimore, Maryland, USA.; 2 Departments of Medicine and Biological Chemistry, Johns Hopkins University School of Medicine, Baltimore, Maryland, USA.; 3 Institute for Fundamental Biomedical Research, Johns Hopkins All Children’s Hospital, St. Petersburg, Florida, USA.

## Abstract

A fundamental and highly contested issue in microbiome research is whether internal organs such as the liver, brain, placenta, pancreas, and others are sterile and privileged or harbor a detectable and functional microbial biomass. In this issue of the *JCI*, Leinwand, Paul, et al. addressed this question using an array of diverse techniques and reported that normal healthy liver possesses a microbiome that is selectively recruited from the gut. They further showed that liver-enriched microbes contributed to shaping the immune network of this organ. Here, we attempt to put their findings into the context of other organs, discuss the technical challenges of defining such microbial communities, and provide some perspective about the road ahead for the field.

## Microbe communities associated with the animal and human body

All animals, including humans and mice, have a complex relationship with microbes. This complexity spans the spectrum of symbioses and includes beneficial, neutral, and pathogenic interactions for the host. The nature of the interaction depends on the context of the encounter, which is heavily influenced by the physiological or disease state of the host. For example, under homeostatic conditions, the gut microbiome, the most dense and diverse community of microbes associated with the human body, has a largely beneficial role, contributing to host metabolism, producing essential metabolites, shaping the immune system, etc. (1, 2). However, for some disease states, such as inflammatory bowel disease (IBD), microbiome composition is altered and can contribute to the pathogenic processes, such that restoring a healthier gut microbiome can be therapeutically beneficial in IBD (3). In nonalcoholic steatohepatitis (NASH), the gut vascular barrier (GVB), a key gatekeeper of bacterial infiltration into internal organs under homeostatic conditions, is damaged, leading to the translocation of bacteria and their products (4). The nature of these dynamically changing relationships, the boundaries between health and disease, and thus the boundaries between bacteria and mammalian organs have been the subject of intense investigation over the past two decades, leading to important insights as to how these interactions might shape host immune responses (5).

An area of interest, controversy, and debate has been in assessing whether internal organs connected to internal and external surfaces via blood and lymphatic vessels contain a functional microbiome or remain sterile and/or privileged under homeostatic conditions.

## Bacteria in the liver

In this issue of the *JCI*, Leinwand, Paul, and colleagues (6) studied the bacterial composition of the liver, using mouse models and human tissue samples. Employing a variety of techniques, they showed that, contrary to conventional wisdom, the liver possessed a small but consistently detectable microbial biomass that is selectively populated from the gut. This community was dominated by *Bacteroidetes*, but shifted dynamically with age and by sex and appeared to have a decisive role in shaping the immune cell composition and response of the liver.

**Figure 1:**
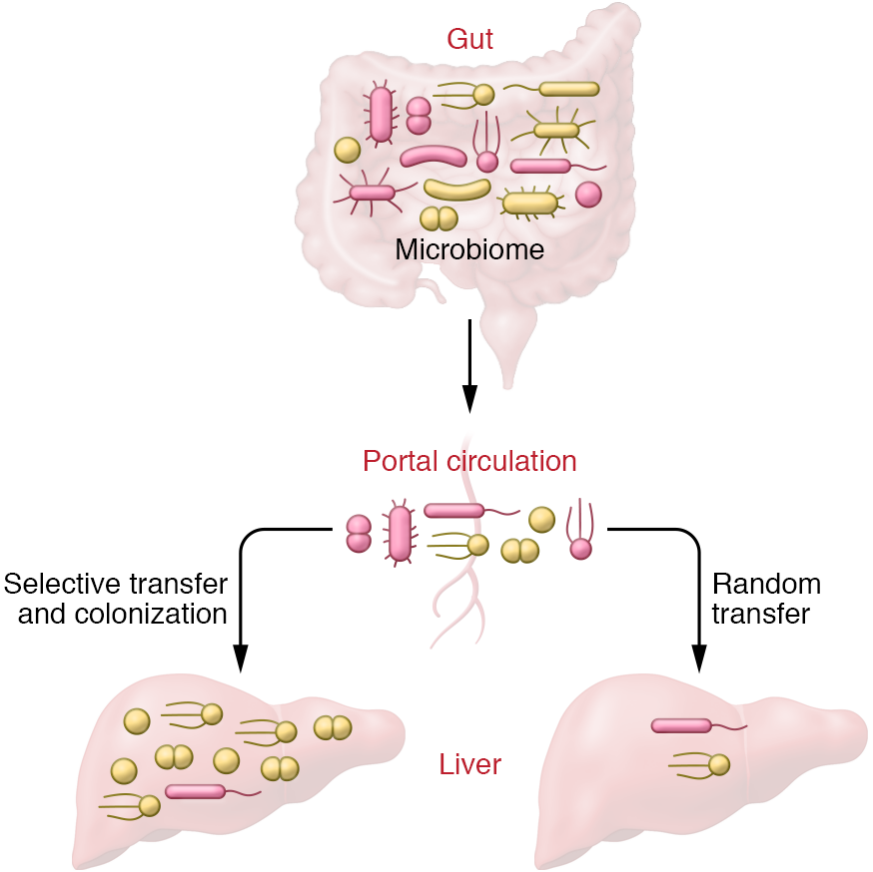
Two scenarios explain the presence of bacteria in healthy liver. A microbiome may establish in the liver through the portal circulation, due to infiltration and colonization by bacterial species suited to this environment. Alternatively, the infiltration of bacteria into the liver is sporadic and occurs via random transfer of bacteria from the gut via the portal circulation, leading to a transient microbe association.

Given prevailing dogma that the liver is a sterile organ, establishing the existence of a functional bacterial niche in such an organ is a challenge that necessarily requires a high bar of supporting evidence. Optimally, one needs to show not only that the bacteria are detectable and stable over time, but also that they are alive, which ideally means they can be cultured and are metabolically active. For the liver, meeting such criteria is particularly challenging, since this organ receives the portal circulation, which drains the gut and may contain microbes that translocate as part of normal physiological processes (Figure 1). Hence, the possibility of separating liver-residing bacteria that would constitute a true organ-associated microbiome from trapped bacteria carried in the portal blood flow is a daunting experimental problem.

Leinwand, Paul, and colleagues go a long way to meet such criteria, but not necessarily all the way. It is not a criticism, since all scientific work by its nature is incomplete. Rather, we would like to point out that the evidence bar is high and establishing accurate data is difficult. The researchers detected a microbial population in the liver by quantitative PCR (qPCR) using targeted 16S rRNA primers and employed a full community analysis in normal mouse liver using 16S rRNA high-throughput sequencing. Of the bacteria detected, 50% were Bacteroidetes, and 25% and 20% were Firmicutes and Proteobacteria, respectively. Interestingly, compared with bacteria in the gut, Proteobacteria were greatly enriched (40-fold). Differences in hepatic and gut bacterial composition could support the hypothesis of a defined liver microbiome, but such differences could also reflect selective transfer process for certain bacterial species or simply better survival of these species in the hepatic tissue. In characterizing the normal liver microbiome in mice, Leinwand, Paul, et al. observed it changing in both an age- and sex-specific manner. Importantly, they also detected bacteria from human liver, including using microscopy to observe intact, live cells. Regarding the dynamic nature and the function of the detected microbiome, they showed that antibiotic treatment could modulate the composition of the liver microbiome and that it also changed in chronic liver fibrosis and upon acute acetaminophen-induced injury. Finally, they provided correlative evidence that the liver microbiome, and in particular *Bacteroidetes* spp., were important for the expansion of liver immune cell numbers and cell types, for example, invariant NKT cells via CD1d and CCL5. However, the functional link to bacterial glycolipids required for CD1d remains elusive. It was unclear how selective bacterial transfer occurred from the gut and how intraorgan bacteria avoided immune surveillance. There were also technical issues regarding the extreme sensitivity of sequencing, particularly from low microbial biomass samples, appropriate experimental controls, and the impossibility of establishing absolute sterility of the liver. These issues would need to be addressed and resolved in future studies.

## The way forward

This is not the first attempt to determine whether sterile organs harbor a microbiome. The same investigators recently reported on the microbiome of the pancreas (7). In a diversity of insects, there is growing appreciation that sites once thought to be sterile, such as the blood (hemolymph) and brain, can be stably or transiently associated with nonpathogenic microbes, despite robust immune mechanisms believed to limit such microbes (8). Yet as these studies expand, a cautionary note is provided by lessons learned from the controversy around the placental/prenatal microbiome (9). In initial studies, the use of powerful and sensitive high-throughput sequencing documented the presence of bacteria in samples of healthy placenta, amniotic fluid, meconium, and even fetal tissues, challenging the dogma of sterility of the womb (9). However, several subsequent studies challenged these results, identifying contamination as the likely source of the findings (10, 11). By analogy, the existence of a brain microbiome has been suggested, and numerous papers have reported the detection of microbial sequences or epitopes in normal and also pathological human brain samples (12). While the evidence from these studies regarding the presence of microbes in diseased brain tissue appears strong, more work is needed to convincingly establish the existence of bacteria in the healthy brain (12). At the same time, the boundaries between health and disease states, whether in the brain, placenta, or liver, are not easy to define. Together, these observations lead to academic debate and highlight the need for modern guidelines to fulfill Koch’s postulates when defining microbiomes in organs previously considered sterile (13, 14). The interesting and provocative work by Leinwand and Paul et al. (6) is another exciting chapter on microbiome-host interactions, but certainly not the last word.

## Version 1 04/15/2022

Electronic publication

## References

[1] Mohajeri MH (2018). The role of the microbiome for human health: from basic science to clinical applications. Eur J Nutr.

[2] Sender R (2016). Are we really vastly outnumbered? Revisiting the ratio of bacterial to host cells in humans. Cell.

[3] Glassner KL (2020). The microbiome and inflammatory bowel disease. J Allergy Clin Immunol.

[4] Mouries J (2019). Microbiota-driven gut vascular barrier disruption is a prerequisite for non-alcoholic steatohepatitis development. J Hepatol.

[5] An D (2014). Sphingolipids from a symbiotic microbe regulate homeostasis of host intestinal natural killer T cells. Cell.

[6] Leinwand J (2022). Intrahepatic microbes govern liver immunity by programming NKT cells. J Clin Invest.

[7] Pushalkar S (2018). The pancreatic cancer microbiome promotes oncogenesis by induction of innate and adaptive immune suppression. Cancer Discov.

[8] Blow F, Douglas AE (2019). The hemolymph microbiome of insects. J Insect Physiol.

[9] Blaser MJ (2021). Lessons learned from the prenatal microbiome controversy. Microbiome.

[10] de Goffau MC (2019). Author Correction: human placenta has no microbiome but can contain potential pathogens. Nature.

[11] de Goffau MC (2019). Human placenta has no microbiome but can contain potential pathogens. Nature.

[12] Link CD (2021). Is there a brain microbiome?. Neurosci Insights.

[13] Salter SJ (2014). Reagent and laboratory contamination can critically impact sequence-based microbiome analyses. BMC Biol.

[14] Amos GCA (2020). Developing standards for the microbiome field. Microbiome.

